# Light-enhanced incoherence of electronic transport in quantum cascade lasers

**DOI:** 10.1038/s41598-020-66302-4

**Published:** 2020-06-09

**Authors:** Andrzej Kolek

**Affiliations:** Department of Electronics Fundamentals, Rzeszów University of Technology, al. Powstańców Warszawy 12, 35-959 Rzeszów, Poland

**Keywords:** Lasers, LEDs and light sources, Electronics, photonics and device physics

## Abstract

Since their invention in the middle of the 1990s, quantum cascade lasers (QCLs) attract increasing theoretical interest stimulated by their widening applications. One of the key theoretical issues is the optimization of electronic transport which in most of these devices is governed by the injection barrier of QCL heterostructure. In the paper, the nonequilibrium Green’s function formalism is used to study electronic transition through the injection barrier as a function of laser field in the cavity; for the increasing field, a crossover is observed from the strong coupling regime, in which electronic transport through the barrier is coherent, to the weak coupling regime, in which electronic transport gets incoherent. This crossover is characterized by gain recovery time, *τ*_rec_, which takes sub-picosecond values for mid-IR QCLs operating at room temperature. This time is also important for the performance of devices under steady-state conditions; the maximum output power is obtained when the figure of merit, FOM = (*g*(0)/*g*_th_ − 1)/*g*_c_*τ*_rec_ [*g*(0) is the linear response gain, *g*_th_ is the threshold gain needed to compensate all losses, *g*_*c*_ is the gain cross-section], reaches maximum. It is shown that the use of this optimization criterion can result in the structures essentially different from those which can be obtained when the optimized quantity is the linear response gain, *g*(0).

## Introduction

Quantum cascade laser (QCL) is a semiconductor device used to emit the radiation in the mid-IR or THz frequency range. Opposed to interband devices, it relays on the intersubband transitions occurring exclusively in the conduction band^1^. The population inversion between the laser subbands, which is necessary for the laser action, arises due to the accumulation of electrons in the upper laser subband and very fast extraction of these electrons from the lower laser subband. The upper subband is fed by the electrons tunneling resonantly from the injector through the injection barrier. The QCL emitting in the mid-IR range usually works in the so-called *strong-coupling* regime^2^. This description refers to the coupling of electronic states located on the opposite sides of the injection barrier; namely: the injector ground state and the upper laser state. When these states are strongly coupled, the carrier transport through the injection barrier is coherent; the nonstationary superposition of the wavefunctions of the states involved in the tunneling transition forms a wave-packet which performs several oscillations before its phase is lost due to the inelastic out-scattering from the upper laser state^3–6^. Then, the transition through the barrier can be considered as low-impedance if compared to the upper-to-lower transition caused by the inelastic out-scattering. Consequently, both states involved in the tunneling transition are approximately equally populated, and the current is limited by the inelastic scattering^2^. This is equivalent to the assumptions made in the semi-classical description of electronic transport^7,8^. Therefore, it is believed that the methods relying on the Boltzmann transport equation, like rate equation (RE) or Monte Carlo (MC), are sufficient for realistic modeling of mid-IR QCLs^3,7^. The situation may change when the interaction with electromagnetic (e-m) field occurs. For the wavelengths corresponding to lasing transition, the radiative out-scatterings from the upper state become more frequent and may push the device into the *weak-coupling* regime where electronic transport is incoherent. In this regime, electrons tunneling from the injector lose their phase before they reach the upper lasing state, and so the tunneling transition becomes a limiting factor. The maximum current that can flow in the structure is reduced, and so the optical power. To prevent this situation, the injection barrier is optimized in such a way that the device remains in the strong coupling regime, even if it lases. The optimization of the barrier is not an easy task because it implies the use of the model that works well both in weak and strong coupling regimes. Examples that use the density matrix (DM) method were proposed by Dupont *et al*.^9^ for THz QCL and by Khurgin *et al*.^10^ and Dinh *et al*.^11^ for mid-IR devices. In these attempts, the optimization of the injection barrier aims at the maximization of the gain peak value. This value can be reasonably estimated only when a method that can account for the quantum aspects of electronic transport is used. DM is one of such methods; however, it needs some phenomenological dephasing times which - to some extent - are arbitrarily assumed^12^. Then, the results of the optimization made with DM method suffer from this arbitrariness.

In this paper, another method, that equally treats tunneling and scattering, is employed to study the crossover between the coupling regimes in mid-IR QCL forced by the interaction with the e-m field. This method uses the nonequilibrium Green’s functions (NEGF) theory which was formulated in the 1960s^13,14^, and since then has been successfully applied to many transport problems, including the ones in QCLs^15–17^. When using the NEGF method, the phase-breaking time does not need to be arbitrarily assumed, or speculated from other considerations, because it inherently arises as a result of the interplay between coherent (quantum tunneling) and incoherent (scattering) processes. Quantities related to this time, like linewidth of gain/absorption spectrum, are then reasonably estimated and do not depend on arbitrarily assumed parameters. In this study, the NEGF method is applied to the number of real QCL structures emitting light of ~5.2 μm wavelength that were tailored for the use in the systems of nitric oxide detection. It is shown that some of these, otherwise very efficient devices, suffer from potential incoherence latent in their structures.

## QCL modeling

### Density matrix model

**Figure 1:**
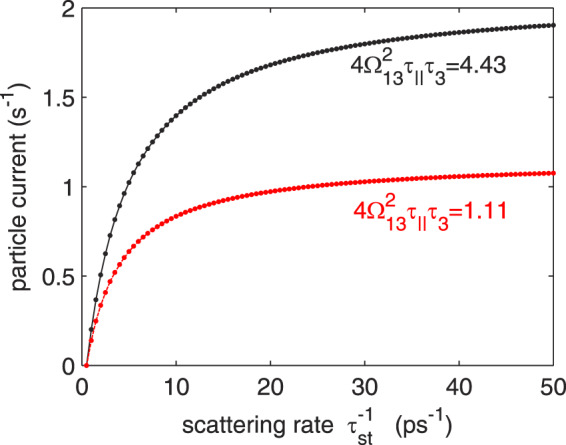
Maximum current increase, *j*(Φ) − *j*(0), as a function of stimulated emission rate 1/*τ*_st_ in the 3-state systems with different coupling energies: ${\hslash \Omega }_{13}=2\,{\rm{m}}{\rm{e}}{\rm{V}}$ (lower) or 4 meV (upper).

The issues raised in the *Introduction* can be illustrated with a simple 3-state density matrix model, in which the injector state (no. 1) with energy ${E}_{1}$ and upper laser state (no. 3) with energy ${E}_{3}$ are resonantly coupled by the injection barrier. The strength of this coupling is given by the coupling energy ${\hslash \Omega }_{13}$. The carriers are out-scattered from the upper state either to the lower laser state (no. 2) with the rate 1/*τ*_32_ or directly to the injector state of the next stage of the cascade with the rate 1/*τ*_31_. State 1 can also be populated from state 2 with the rate 1/*τ*_21_. Apart from these *non-radiative* channels, a *radiative* channel for $3\to 2$ transition can be opened by the laser field in the cavity. If the population inversion, $\Delta {n}_{32}={n}_{3}-{n}_{2}$, between the populations *n*_*i*_ in states 3 and 2 goes to positive values, the flux of electrons flowing in this channel is given by the product of gain cross-section *g*_*c*_, photon flux density Φ (per unit active region width), length *d* of one QCL module, and $\Delta {n}_{32}$. The first three define the field-dependent stimulated emission rate, $1/{\tau }_{{\rm{st}}}={g}_{c}d\Phi$. In this model, the spontaneous emission (much lower than the stimulated one) is not accounted for. When periodic boundary conditions are imposed and the total population in all states is normalized to unity, the steady-state particle current flowing through such system is given by

1 $$j=i{\Omega }_{13}({\rho }_{31}-{\rho }_{13})=\frac{1}{{T}_{i}+\frac{2{\tau }_{32}+{\tau }_{21}+3{\tau }_{32}{\tau }_{21}/{\tau }_{{\rm{st}}}}{1+{\tau }_{32}/{\tau }_{31}+{\tau }_{32}/{\tau }_{{\rm{st}}}(1+{\tau }_{21}/{\tau }_{31})}}\equiv \frac{1}{{\tau }_{{\rm{tr}}}},$$

where ${\rho }_{ij}$ are the elements of the density matrix, *τ*_tr_ is the effective transport time, and

2 $${T}_{i}\equiv \frac{1+{\Delta }^{2}{\tau }_{\parallel }^{2}}{2{\Omega }_{13}^{2}{\tau }_{\parallel }}$$

is the tunneling time^18^. In Eq. (2), *τ*_||_ denotes the dephasing time at the injection barrier, and $\Delta =({E}_{1}-{E}_{3})/\hslash$ is the detuning from the resonance of 1–3 doublet. The mentioned arbitrariness of the DM model is stored in the time *τ*_||_; ${\tau }_{\parallel }^{-1}$ is half the sum of the state decaying rates due to intersubband processes plus some “pure” dephasing rate, which is a phenomenological component^4^. Substituting Eq. (2) into Eq. (1), one gets the well-known Kazarinov-Suris relation^19^ in a form adapted for the current 3-state system

3 $$j=\frac{2{\Omega }_{13}^{2}{\tau }_{\parallel }}{1+{\Delta }^{2}{\tau }_{\parallel }^{2}+4{\Omega }_{13}^{2}{\tau }_{3}^{\ast }{\tau }_{\parallel }},$$

where ${\tau }_{3}^{\ast }={\tau }_{{\rm{tr}}}-{T}_{i}$. For the lasing device, the amount of the current increment, $j(\Phi )-j(\Phi =0)$, caused by the laser field is of interest, because it is directly related to the flux of particles that can emit the photon and so to the output power. In Fig. 1, this quantity is plotted using Eqs. (1) and (2) for two values of the coupling energy: ${\hslash \Omega }_{13}=2$ meV or 4 meV, and the values of other parameters: ${\tau }_{32}=1\,{\rm{ps}}$, ${\tau }_{31}=4\,{\rm{ps}}$, ${\tau }_{21}={\tau }_{\parallel }=0.15\,{\rm{ps}}$, reasonable for mid-IR QCL. The calculations were done at the resonance, i.e., for Δ = 0, so the plots represent the maximum increase of the current and the maximum optical power available in the system.

**Figure 2:**
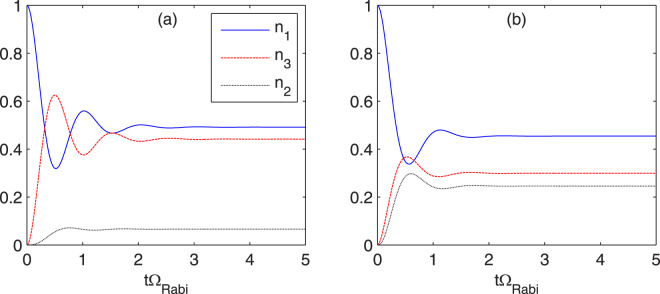
Time evolution of states occupation after short pumping of the injector state 1, calculated for Δ = 0 and typical designing values of mid-IR QCL: ${\hslash \Omega }_{13}=4\,{\rm{m}}{\rm{e}}{\rm{V}}$, *τ*_32_ = 1 ps, *τ*_31_ = 4 ps, *τ*_21_ = *τ*_||_ = 0.15 ps for the case: (**a**) without, (**b**) with the laser field characterized by the stimulated emission time, *τ*_st_ = 40 fs. Time *t* on the horizontal axis is scaled by the Rabi frequency (for the tunneling transition), ${\Omega }_{{\rm{Rabi}}}=\sqrt{4{\Omega }_{13}^{2}+{\Delta }^{2}}/2\pi$.

The coupling regimes are defined by the relation between the terms in the denominator of Eq. (3) ^2^. Namely, the strong coupling (coherent transport) occurs when the last term largely exceeds the first two. Then, initially (i.e., for $\Phi =1/{\tau }_{{\rm{st}}}=0$), the system is in the coherent regime for both values of the coupling energy, although for *ħ*Ω_13_ = 2 meV the system is “less coherent”, i.e., it has lower value of ${\hslash \Omega }_{13}^{2}{\tau }_{3}^{\ast }{\tau }_{\parallel }$. In the strong coupling regime: the current *j* is exclusively described by the scattering lifetime ${\tau }_{3}^{\ast }$, the occupations $({n}_{i}={\rho }_{ii})$ of states 1 and 3 are approximately equal and - as illustrated in Fig. 2a - electrons perform several oscillations before they settle at the steady-state value. As shown in Fig. 2b, the coherence is destroyed by the laser field; in this case, the oscillations are more damped and disappear after one cycle. As demonstrated in Fig. 1, the current increases with the laser field; however, the increase of the current is lower for initially less coherent system than for the other one. Worth to note is that both oscillatory and non-oscillatory responses were observed in the number of pump-probe experiments^5,6,20,21^.

With this DM model, the population inversion can be calculated as $\Delta {n}_{32}={\rho }_{33}-{\rho }_{22}$ and occurs to depend on the dephasing time, *τ*_||_. The inversion gain, $g={g}_{c}\Delta {n}_{32}$, also depends on this time, which one would like to avoid as its value comprises phenomenological components. The NEGF model presented in the next section does not have such disadvantage.

### NEGF model

In the NEGF approach, the dephasing time does not appear *per se*. States and gain broadenings arise exclusively from all inelastic and elastic scatterings included in the model as well as due to the fact that the quantum system is open. All these factors are taken into account through theoretically well-justified physical models: the system Hamiltonian and the scattering selfenergies. As the aim is to derive conclusions for real QCL structures, the NEGF model of the device presented in this section is much more realistic than the simple 3-state system considered in the previous section.

So far, all QCL nanostructures are n-type. They consist of parallel layers made of different materials. The appropriate Hamiltonian is then one-band 1D operator

4 $$H=\frac{-\,{\hslash }^{2}}{2}\frac{d}{dz}\frac{1}{m(E,z)}\frac{d}{dz}+V(z)+\frac{{\hslash }^{2}{k}^{2}}{2m(E,z)}$$

in the growth direction *z*, parametrized for the in-plane momentum modulus, $k={k}_{xy}$^1,16^. In Eq. (4), the potential $V(z)$ includes both the variation of the conduction band edge *E*_*c*_(*z*) and the Hartree potential. Nonparabolicity is accounted for through the energy-dependent effective mass, $m(E,z)={m}^{\ast }(z)[1+(E-{E}_{c}(z))/{E}_{g}(z)]$, where *E*_*g*_ is the bandgap energy. Other symbols have the usual meaning. It was shown that such Hamiltonian gives results that very well match the results obtained with the eight-band kp model^1^.

The equations of the NEGF formalism^13,14^ applied to the Hamiltonian of Eq. (4) were solved numerically in the real space (position basis) for the steady-state. The scattering selfenergies in the formulations, suitable for the Hamiltonian of Eq. (4), were provided in the number of papers^16,22,23^. They cover most important scattering processes involving: optical and acoustic phonons, ionized impurities, alloy disorder, and interface roughness. Recently, the selfenergies for the interaction with the optical field have been also provided^24^, what enables this study. As already mentioned, the electron-electron interactions were treated within the mean-field approximation. This was done by solving the Poisson equation self-consistently with the NEGF equations. For the former, the boundary conditions ensured device neutrality. At the output of the procedure, the 4-argument Green’s functions $G(z,z{\prime} ,E,k)$ were obtained. Physical quantities were calculated from functions *G*^*R*^ and *G*^<^. Appropriate formulas for the densities of currents, states, and carriers are provided, e.g., in ref. ^16^. The linear response (small signal) gain/absorption was calculated based on the theory outlined in ref. ^15^, adapted for the case of energy-dependent effective mass^25^. More details of NEGF calculations are provided in the *Methods* section.

### Devices

The attention is paid to two QCL structures that emit IR radiation at ~5.2 μm wavelength. Namely: structure D of Diehl *et al*.^26^, and structure E of Evans *et al*.^27^. Both structures are very efficient. However, the output power of ~0.3 W emitted at room temperature from structure D is only approximately half of that emitted from structure E under similar operating conditions. We claim that the poorer performance of structure D is due to the larger incoherence of tunneling transition forced by the laser field in this structure. To prove this claim, the simulations were performed; however not for original devices. For those used in the simulations, all parameters, which can influence the output power, were kept identical. Consequently, the calculations were performed for devices which have equal number of periods, ${N}_{p}=30$, and were doped to the same value of sheet density, ${n}_{{\rm{dop}}}=8.9\times {10}^{10}\,{{\rm{cm}}}^{-2}$, in the injector layers, although different doping profiles proposed by the structures designers were maintained. Also, the same value of the threshold gain, ${g}_{{\rm{th}}}=9.5\,{{\rm{cm}}}^{-1}$, was assumed for both devices. This value corresponds to overall losses: *α*_m_ = 2 cm^−1^, *α*_w_ = 3.8 cm^−1^ in a typical waveguide, with a confinement factor, $\Gamma =0.6$^26^. Under such assumptions, the output optical power was calculated as^28^

5 $$P=(1-R){N}_{p}\,w\,d\,{\Phi }_{{\rm{th}}}\,{E}_{\gamma },$$

where *w* = 7.5 μm is the cavity width, *R* = 0.27 is the facet reflectivity, and *E*_*γ*_ is the photon energy. As *d* and *E*_*γ*_ are very similar for both structures, the output power depends exclusively on the value of the photon flux Φ_th_ required to clamp the gain to its threshold value *g*_th_. In general, Φ_th_ can be different for different structures.

## Results and Discussion

**Figure 3:**
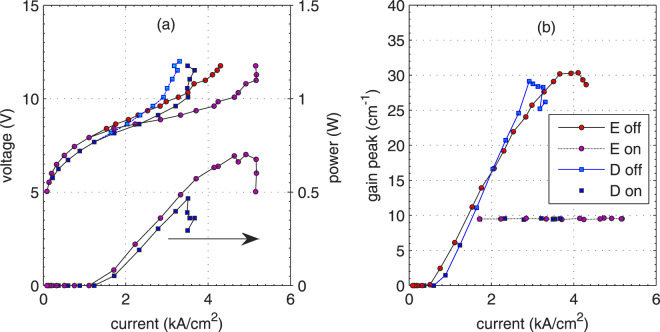
(**a**) Current-voltage and light-current, and (**b**) gain-current characteristics calculated using the NEGF method for structures E and D with (on) or without (off) laser field included in the calculations (through electron-photon selfenergies).

Results of the simulations presented in Fig. 3a confirm the experimental observation; the output power available from structure D is lower by 30–40% than from structure E. The absolute values of the optical power are also not very far from those reported in the experiment, although the differences are expected; real devices have different waveguide dimensions and are doped to different densities. They work continuous wave (cw), and so suffer from self-heating. On the contrary, the simulations assume that the dissipated electrical power does not heat up the structures.

Data in Fig. 3 illustrate how the light-current-voltage characteristics were obtained. Initially, current-voltage (I-V) and gain-current characteristics were calculated for the laser field turned off. In the second step, the laser field was turned on and increased until the gain peak was clamped to the threshold value, ${g}_{{\rm{th}}}=9.5\,{{\rm{cm}}}^{-1}$. Data for the “off” state are also included in Fig. 3. One can observe that the amount of excess current, *I*_on_ - *I*_off_, that can be converted into photon flux is larger for structure E than for structure D. According to the DM model, this can be attributed to more coherent transport in structure E. Indeed, the calculations of the coupling energy performed like in ref. ^29^ give the values: ${\hslash \Omega }_{13}=2.07\,{\rm{m}}{\rm{e}}{\rm{V}}$ for structure D and ${\hslash \Omega }_{13}=3.55\,{\rm{m}}{\rm{e}}{\rm{V}}$ for structure E. The difference is due to different thicknesses of the injection barrier, which is 4.2 nm for structure D and 2.9 nm for structure E.

**Figure 4:**
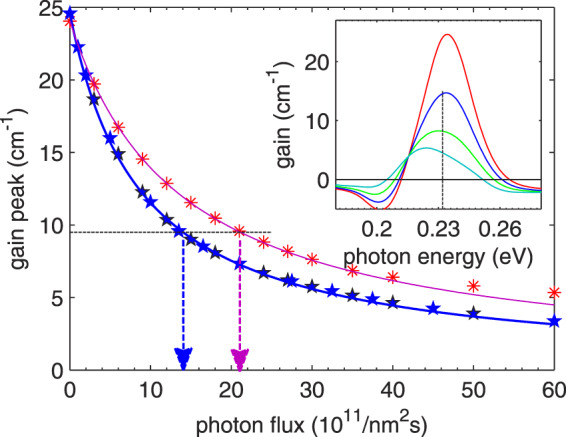
Gain peak calculated with NEGF method (symbols) at constant bias voltage 320 mV/period for structures E (upper) and D (lower) as a function of monochromatic photon flux. Lines are the plots of Eq. (6) drawn for ${g}_{c}=0.64\times {10}^{-8}\,{\rm{cm}}$, ${\tau }_{{\rm{rec}}}=0.23\,{\rm{ps}}$ for structure E or ${g}_{c}=0.60\times {10}^{-8}\,{\rm{cm}}$, ${\tau }_{{\rm{rec}}}=0.37\,{\rm{ps}}$ for structure D. Inset: gain spectra calculated for structure E for the increasing (up to down) monochromatic photon flux of photons with the energy *E*_*γ*_ = 232 meV (dashed line). For the highest optical fields, gain bleaches and gain peak moves aside the photons’ energy *E*_*γ*_. As neither of these effects is included in the DM model, the deviations between the simulations and the plot of Eq. (6) are observed in the main figure for structure E.

One can observe in Fig. 3b that for both structures the gain peak in the “off” state reaches approximately the same value of 30 cm^−1^. While lasing, the gain is clamped to the value *g*_th_, which is also equal for both structures. As the increase of the current, *I*_on_ - *I*_off_, is lower for structure D, the suppression of the gain by the optical field must be more “efficient” in this structure. This “efficiency” can be quantified by the *gain recovery time*, *τ*_rec_, appearing in the relation^30^

6 $$g(\Phi )=\frac{g(0)}{1+{\tau }_{{\rm{rec}}}\,{g}_{c}\,d\,\Phi },$$

or alternatively by the *saturation intensity*, Φ_s_ = 1/(*τ*_rec_ *g*_*c*_ *d*)^31^. According to the DM model

7 $${\tau }_{{\rm{rec}}}={\tau }_{21}+{\tau }_{{\rm{eff}}}\frac{{T}_{i}+{\tau }_{21}}{{T}_{i}+3{\tau }_{3}-{\tau }_{{\rm{eff}}}},$$

where ${\tau }_{3}={\tau }_{32}{\tau }_{31}/({\tau }_{32}+{\tau }_{31})$ is the upper state lifetime and ${\tau }_{{\rm{eff}}}={\tau }_{3}(1-{\tau }_{21}/{\tau }_{31})$ is the effective upper state lifetime. Only in the case ${T}_{i}\to \infty$, which is equivalent to very weak coupling, the gain recovery time *τ*_rec_ can be written in the form predicted by the semi-classical RE approach, ${\tau }_{{\rm{rec}}}={\tau }_{21}+{\tau }_{{\rm{eff}}}$^30^. For the structures in question, the gain recovery time can be evaluated from the data presented in Fig. 4. In the simulations made for this purpose, the bias voltage was kept at a constant value (in order to fix the tunneling time), while the photon flux was increased and gain spectrum was calculated for certain values of the photon flux. In general, the dependence observed in Fig. 4 demonstrates the decrease of the gain caused by the optical field. As can be seen, the gain suppression is stronger for structure D, as expected for “less coherent” structure. The curves differ and larger flux is needed in order to clamp the gain to the threshold value for structure E. Therefore, this structure emits more power. Gain recovery time estimated by fitting the data in Fig. 4 to Eq. (6) takes the value ${\tau }_{{\rm{rec}}}=0.34\,{\rm{ps}}$ for structure D and only ${\tau }_{{\rm{rec}}}=0.24\,{\rm{ps}}$ for structure E. The values of gain cross-section necessary to get these estimates were obtained in an independent numerical simulation described in the *Methods* section. The estimates of *τ*_rec_ are a bit lower than the value 0.47 ps found in a similar numerical experiment for another mid-IR QCL^32^. The larger value of the gain recovery time implies that the structure considered in ref. ^32^ was even “more incoherent”. Indeed, the value ${\hslash \Omega }_{13}^{2}\,{\tau }_{3}^{\ast }\,{\tau }_{\parallel }\cong 0.2$ estimated for this structure^32^ classifies it deeply in the weak coupling regime. The observed dependency: the stronger coupling, the shorter gain recovery time suggests that the latter cannot be exclusively connected with the scattering times as predicted by semi-classical models^30,32^. As stems directly from Eq. (7), this time also depends on the tunneling time which is related to the strength of the coupling energy, ${\Omega }_{13}$, through Eq. (2).

## Structure optimization

**Figure 5:**
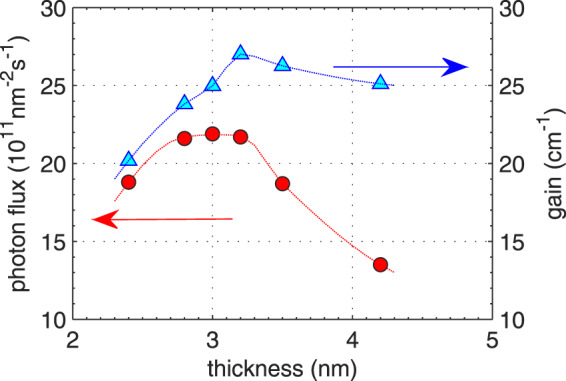
Photon flux Φ_th_ required to clamp the gain to the threshold value ${g}_{{\rm{th}}}=9.5\,{{\rm{cm}}}^{-1}$ (circles - left axis) and gain peak at zero flux $g\mathrm{(0)}$ (triangles - right axis), calculated with the NEGF method for structure D with various injection barrier thicknesses. Structures were biased with the voltage *U* = 320 mV/period.

**Figure 6:**
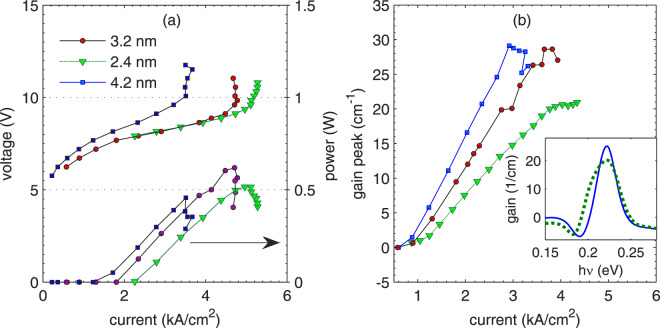
(**a**) Current-voltage and light-current, and (**b**) gain-current characteristics calculated using the NEGF method for structure D with different injection barrier thicknesses. Inset: gain spectra for 4.2 nm (solid line) and 2.3 nm (dots) thick barriers.

The analysis performed in the previous section proves that the difference in the performance of structures D and E results from the degree of coherence (of tunneling injector-to-upper-state transition) latent in these structures. The measure of this performance is the gain recovery time which should be minimized to obtain more optical power. The correct strategy for structures optimization (if the output power is a criterion) is not only to maximize the gain (like in refs. ^9–11^) but simultaneously to minimize *τ*_rec_. Quantitatively, the best method for this would be to maximize the quantity

8 $${\Phi }_{{\rm{th}}}=\frac{g(0)/{g}_{{\rm{th}}}-1}{{\tau }_{{\rm{rec}}}{g}_{c}}\to \,{\rm{\max }}\,,$$

which maximizes the phonon flux required to clamp the gain to the threshold value (and so to maximize the optical power) and appears as a figure of merit. Depending on which parameter is being optimized, this criterion may lead to interesting results. If it is the injection barrier thickness or height, and quantities are expressed in the terms of the 3-state DM model, Eq. (8) takes the global maximum at ${T}_{i}=0$, which corresponds to the very thin/low barrier. In this case, the gain spectrum broadens at the expense of the gain peak^33^. This effect is not accounted for in the DM model. To take it into account, a more detailed quantum model of electronic transport, either DM^29^ or NEGF, is necessary. A DM approach that used gain peak as a criterion (but not Eq. (8)) was presented, e.g., in refs. ^9–11^. In the NEGF method, the value of Φ_th_ can be directly calculated (see Fig. 4) without the need of assuming any phenomenological parameters or even the estimation of the quantities, like *τ*_rec_ or *g*_*c*_. The results of calculations that maximize the figure of merit of Eq. (8) for structure D with different barrier thicknesses are presented in Fig. 5. As can be seen, Φ_th_ goes through a broad maximum which means that finding optimal barrier thickness is possible. Indeed, the maximum output power *P* = 0.62 W found for structures with 3.2 nm-thick barrier is larger than the maximum output power found for structure with 4.2 nm (0.46 W) and 2.4 nm (0.52 W) thick barriers (see Fig. 6). In Fig. 5, the results of optimization, when the linear response gain *g*(0) is the criterion, are also included. As can be seen, they may lead to different conclusions concerning optimal barrier thickness.

## Conclusions

The dependence shown in Fig. 4 demonstrates that the implementation of light-matter interaction in NEGF formalism through appropriate selfenergies enables going beyond linear response to an external e-m field without the need of solving the time-dependent Kadanoff-Baym equation. While the “selfenergy” approach has already been used for interband devices^34–36^, it is new for lasers relying on intersubband transitions. Till now, the light-matter interaction was implemented in the NEGF method applied to QCLs through the decomposition of the Green’s functions and selfenergies into higher harmonics of the lasing frequency^30,32,37^. This approach is more exact, but generates much larger numerical load than the approach presented in the current study.

It was shown that the laser action pushes the structure into the weak coupling regime where transport through the injection barrier is less coherent and limited by tunneling. Numerical simulations provide unique tool to study this light-induced crossover; the upper state lifetime which is a combination of non-radiative and radiative scattering rates can be tuned by changing the photon flux, keeping the remaining parameters unchanged. In working devices, such tuning is hardly possible, because photon flux saturates at the value which clamps the gain to its threshold value. The simulations show that light-enhanced incoherence of the tunneling transition is characterized by the gain recovery time which increases with the decreasing initial (static) coherence latent in the quantum design of the structure. Contrary to semi-classical results, this time depends not only on the scattering lifetimes but also on the tunneling time through the injection barrier. Due to this dependence, the devices (even those emitting in the mid-IR range) may experience the limitations introduced by too long gain recovery time. Real devices suffering from this limitation do exist^26^. To increase their performance, the strategy for barrier optimization should be reconsidered; the criterion of maximizing the linear response gain (gain in the no-lasing state) should be replaced with maximizing the figure of merit provided by Eq. (8). Data presented in Fig. 5 illustrate that these two criteria can lead to different optimal structures. Arriving at these conclusions was possible due to full quantum studies of QCL nanostructures which were done with NEGF method.

## Methods

Results for the DM model were obtained by the analytical solution of the Liouville equation completed with the terms that characterize scattering and dephasing of density matrix elements

$$\frac{d\rho }{dt}=-\,\frac{i}{\hslash }[H,\rho ]-S.$$

For the 3-state model, discussed in the paper, *H*, *ρ* and *S* are 3 × 3 matrices.

The equations of the NEGF formalism in the position basis were adapted from ref. ^16^. They were applied to the structures truncated to a bit more than one QCL module (period). The rest of the cascade was mimicked by suitable contact selfenergies^17^. Non-uniform discretization mesh was used to reduce numerical load without losing the accuracy^38^. The parameters m* and *E*_*g*_ for ternary materials used for QCL layers were taken from ref. ^39^. Conduction bandgap offsets were calculated using model solid theory including strain^40^ and material parameters that were taken from ref. ^41^. This method was also used to evaluate conduction band offsets used in the parametrization of alloy disorder scattering. The scattering selfenergies were implemented in a way described in refs. ^22,23^ for the scatterings from ionized impurities, alloy disorder, LO-phonon, and interface roughness. For scattering from acoustic phonons, the energy-averaged approximation of ref. ^16^ was implemented. The electron-photon selfenergies were calculated using the low-density approximation^24^. All parameters used in the NEGF calculations, as well as the set of microscopic results obtained with the NEGF method for structure D, are provided in the *Supplementary* *Information*.

**Figure 7:**
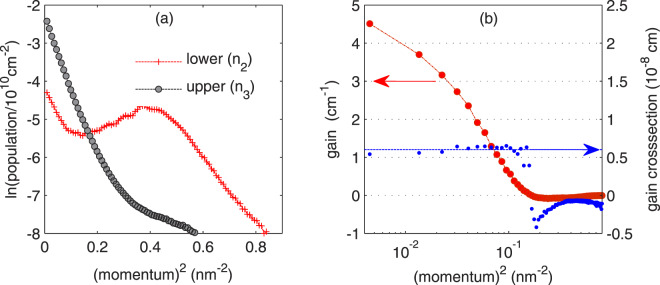
(**a**) Occupations of laser subbands vs. in-plane momentum modulus squared, (**b**) gain (circles) and gain cross-section (dots) at the lasing energy *E*_*γ*_ calculated with the NEGF method for structure D.

The gain cross-section at the lasing energy *E*_*γ*_ can be estimated as the ratio of the gain at this energy and the population inversion, ${g}_{c}({E}_{\gamma })=g({E}_{\gamma })/\Delta {n}_{32}$. This definition may be applied to the total subband populations and total gain only when bands are parabolic and the populations are thermalized. In mid-IR QCLs, energy levels are well above the conduction band edge and nonparabolicity must be taken into account. It was also shown that the populations in QCL subbands are highly nonthermal^42^. In effect, not all carriers contributing to Δ*n*_32_ contribute to *g*(*E*_*γ*_). To get rid of such particles, *g*_*c*_ was estimated from momentum-resolved calculations, ${g}_{c}({E}_{\gamma },k)=g({E}_{\gamma },k)/\Delta {n}_{32}(k)$. The results of these calculations are presented in Fig. 7. Except for the largest momenta, *g*_*c*_ takes an almost momentum-independent value of ~0.60 × 10^8^ cm, quite close to the value 0.57 × 10^−8^ cm found for similar (GaInAs/AlInAs) QCL^28^. At largest *k*’s, *g*_*c*_ drops to lower and negative values since in this range: (i) the gain at the lasing frequency *E*_*γ*_ is not produced (because ${E}_{32}={E}_{3}-{E}_{2} < {E}_{\gamma }$ due to bands nonparabolicity); (ii) the populations are not inverted (see Fig. 7a). For the comparison; if the total (i.e., summed over all $k$) populations in the laser subbands and total gain were used, the (largely) overestimated value, *g*_*c*_ = 1.53 × 10^−8^ cm, was obtained.

## Supplementary information

Supplementary Information.
