# Supplementary material for: Light-enhanced incoherence of electronic transport in quantum cascade lasers
Source: Sci Rep. 2020 Jun 9;10:9318. doi: 10.1038/s41598-020-66302-4 (PMC7283320; doi:10.1038/s41598-020-66302-4)
Supplement: Supplementary file 1 — Supplementary Information. [file 41598_2020_66302_MOESM1_ESM.pdf]

Light-enhanced incoherence of electronic transport in quantum cascade lasers

Andrzej Kolek

Department of Electronics Fundamentals, Rzeszów University of Technology,
Al. Powstańców Warszawy 12, 35-959 Rzeszów, Poland, akoleknd@prz.edu.pl

SUPPLEMENTARY INFORMATION

Supplementary Table 1. Parameters used in NEGF modeling.

	Structure D		Structure E	
	Well	Barrier	Well	Barrier
	$In_{0.6}Ga_{0.4}As$	$Al_{0.56}In_{0.44}As$	$In_{0.66}Ga_{0.34}As$	$Al_{0.654}In_{0.346}As$
m*	0.043	0.085	0.0452	0.0913
E_g (eV)	0.808	1.72	0.8353	1.84
alloy matrix element (eV)	0.42	1.53	0.42	1.53
ΔE_c (eV)	0.67		0.825	
n_{dop} (cm^{-2})	8.9×10^{10}			
LO-phonon energy (eV)	0.032			
deformation potential (eV)	5.89			
density (kg/m3)	5590			
sound velocity (m/s)	4810			
$\varepsilon_0/\varepsilon_\infty$	13.73/11.36			
Δz_{max} (nm) [ref. 37]	0.8			
temperature (K)	288			
τ_{lead} (ps) [ref. 16]	0.1			
N_{per}	30			
interface roughness	Gaussian correlation function			
height (nm)	0.15 (half of monolayer width)			
radius (nm)	9**			

**this value: (i) falls well within the experimental range 6 - 20 nm (see [1] for discussion); (ii) was shown to best fit the measured electroluminescence with the calculated gain spectrum for GaAs/AlGaAs device [2]; (iii) was assumed in DM modeling of InGaAs/AlGaAs QCLs [3][4]; (iv) gives the value of height x radius product close to experimental value 1.01 nm^2 reported for InGaAs/AlGaAs interfaces [5].

[1] M. Franckié, D. O. Winge, J. Wolf, V. Liverini, E. Dupont, V. Trinité, J. Faist, and A. Wacker, "Impact of interface roughness distributions on the operation of quantum cascade lasers," Optics Express 23(4), 5201-5212 (2015). https://doi.org/10.1364/OE.23.005201

[2] M. Bugajski, P. Gutowski, P. Karbownik, A. Kolek, G. Hałdaś, K. Pierściński, D. Pierścińska, J. Kubacka-Traczyk, I. Sankowska, A. Trajnerowicz, K. Kosiel, A. Szerling, J. Grzonka, K. Kurzydłowski, T. Slight, and W. Meredith, "Mid-IR quantum cascade lasers: Device technology and non-equilibrium Green's function modeling of electro-optical characteristics," Phys. Status Solidi (b) 251, 1144 (2014). https://doi.org/10.1002/pssb.201470135

[3] R. Terazzi, and J. Faist, "A density matrix model of transport and radiation in quantum cascade lasers," New J. Phys. 12, 033045 (2010). https://doi.org/10.1088/1367-2630/12/3/033045

[4] M. Lindskog, J. M. Wolf, V. Trinite, V. Liverini, J. Faist, G. Maisons, M. Carras, R. Aidam, R. Ostendorf, and A. Wacker, "Comparative analysis of quantum cascade laser modeling based on density matrices and non-equilibrium Green's functions," Appl. Phys. Lett. 105, 103106 (2014). https://doi.org/10.1063/1.4895123

[5] S. Tsujino, A. Borak, E. Müller, M. Scheinert, C. V. Falub, H. Sigg, D. Grützmacher, M. Giovannini, and J. Faist, "Interface-roughness-induced broadening of intersubband electroluminescence in p-SiGe and n-GaInAs⁄AlInAs quantum-cascade structures," Appl. Phys. Lett. 86, 062113 (2005). https://doi.org/10.1063/1.1862344

Microscopic results obtained with NEGF method

The figures below were obtained for the structure D, described in the paper L. Diehl et al, *Appl. Phys. Lett.* **89**(8), 081101 (2006), biased with the voltage 320 mV/period. All parameters used in the calculations are listed in Table S1.

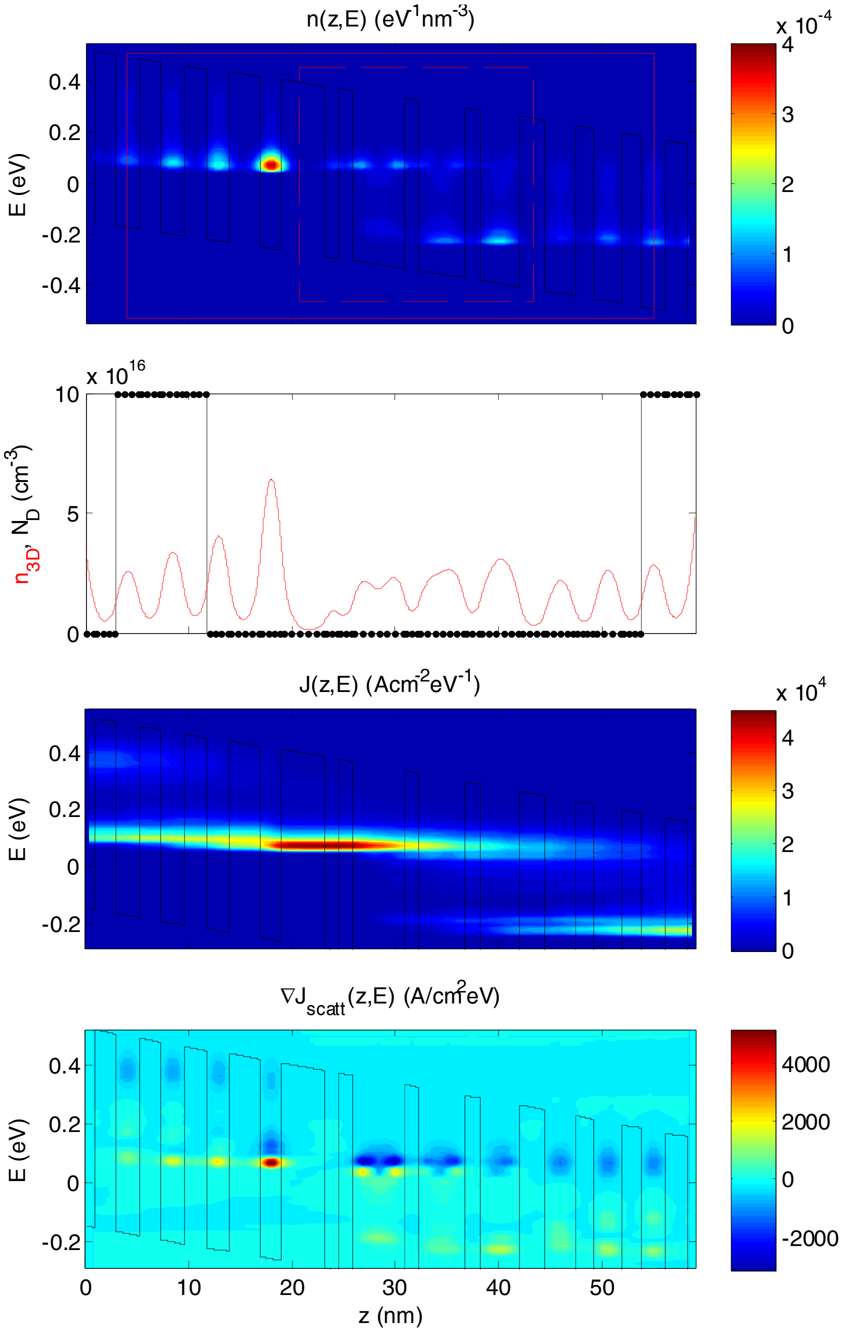

Supplementary figure 1. Position-resolved quantities (upper to lower): electron density (energy-resolved), 3D electron (n_{3D}) and doping (N_D) densities (integrated), current density J, divergence of scattering current ∇J_{scatt}. Lines show conduction band profile. Symbols in the plot of doping density are placed at the discretization points. Boxes in the most upper figure include one QCL period (solid) and light amplification (active) region (dashed).

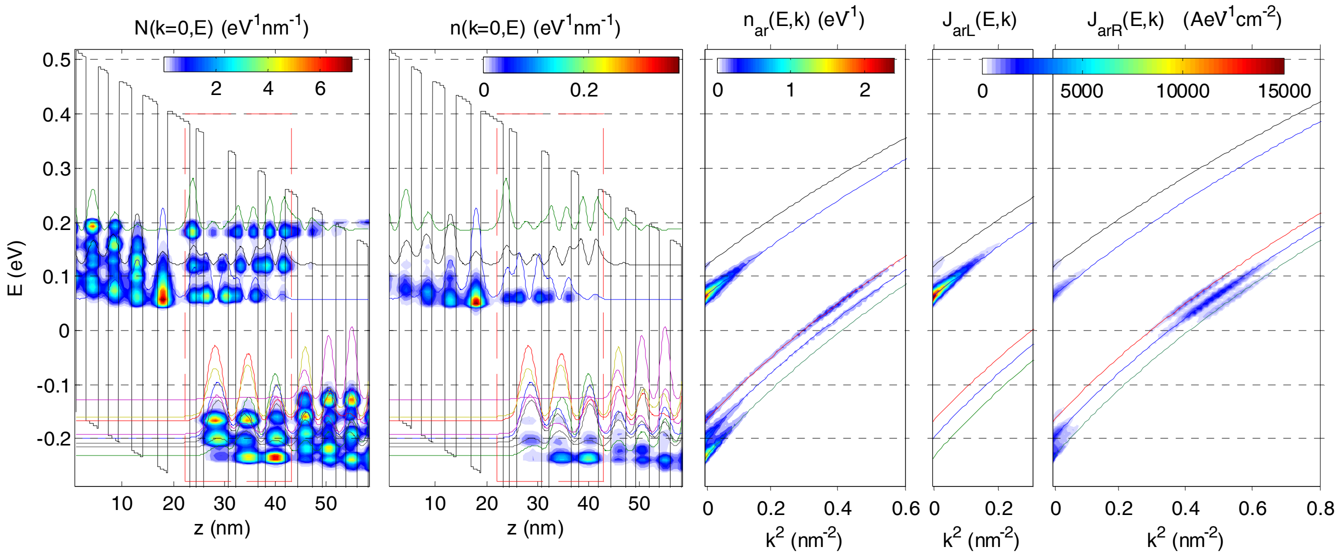

Supplementary figure 2. Position-energy-momentum-resolved quantities: (left to right) density of states, $N(k=0,E,z)$, for vanishing in-plane momentum k, same but for electrons, $n(k=0,E,z)$, density of electrons in active region, $n_{ar}(E,k) = \int_{z\in ar} n(E,k,z)dz$, current density $J_{arL}(E,k)$ at z = arL, i.e., entering active region (dashed-line red box) from the left, same but for z = arR, i.e., leaving active region to the right. Lines show most important states in the position-resolved plots or the subbands in active region in the momentum-resolved plots. The departures from linear k^2 dependence are due to nonparabolicity.

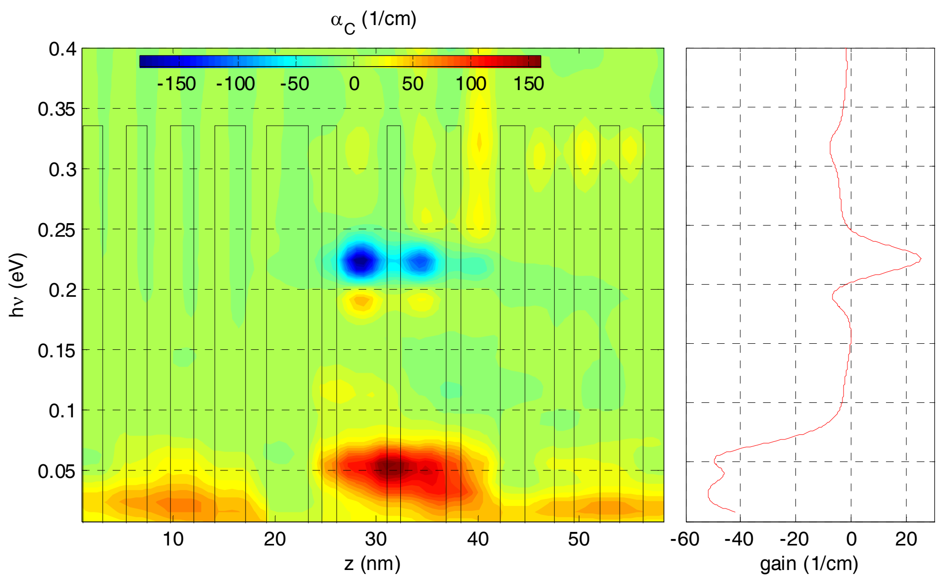

Supplementary figure 3. Frequency-position-resolved absorption coefficient (left) and gain spectrum per period (right).

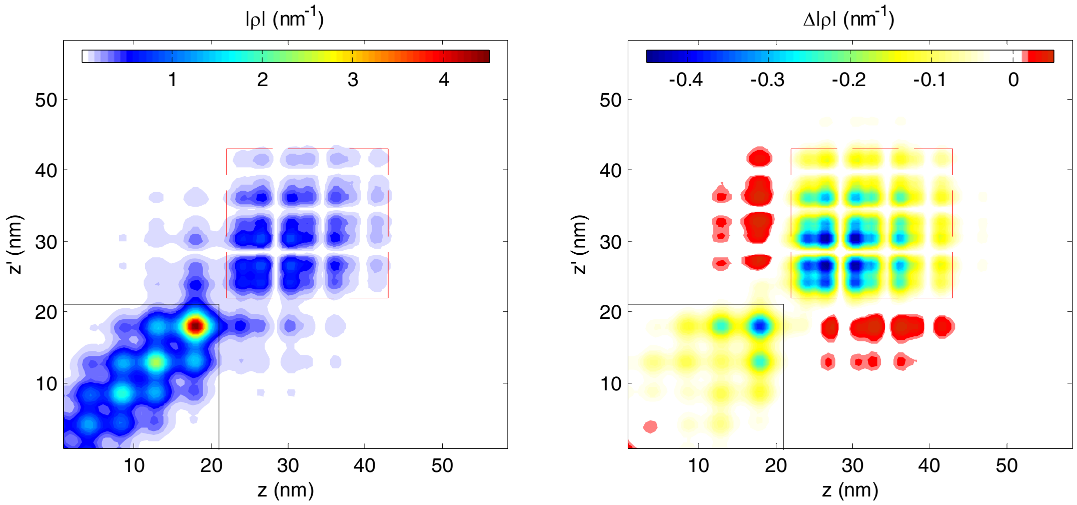

Supplementary figure 4. (left) Density matrix for k = 0, ρ(z, z', k = 0), calculated from lesser Green's function $G^<(z, z', E, k = 0)$ by integration over energies E > 0, so it contains only the coherences between the left injector and the upper laser state. Boxes on diagonal refer to coordinates z, z' inside the injector (solid black) or in the active region (dashed red). Large coherences arise when two states have considerably spatial overlap and a small energy difference. Inside the boxes, states are extended and strongly overlap and so have large coherences. Elements outside the boxes show coherences between the injector states and the upper state. (right) Change of density matrix elements caused by the interaction with optical field (flux 4.2×10^{12} photons/s/nm^2). Densities (diagonal elements) decrease and so intra-box coherences. Inter-box, i.e., injector-active region coherences increase. Such changes are consistent with 3-state DM model (see figure below).

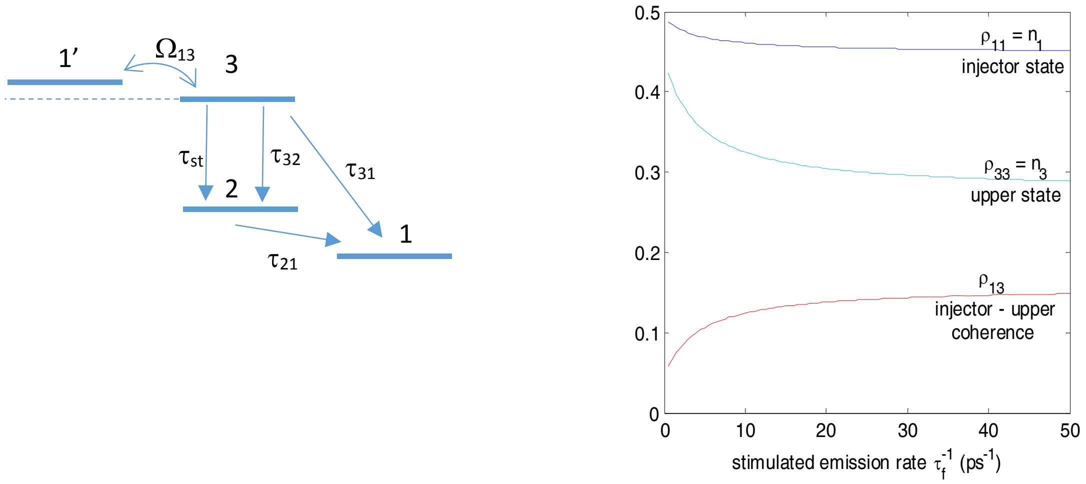

Supplementary figure 5. (left) States and transitions considered in 3-state (density matrix) DM model. (right) Change of DM elements caused by the increasing optical field calculated for 3-state DM model; $1/\tau_{st}$ is the stimulated emission rate which increases with photon flux density.
